# Fluorescence-Based Real-Time Analysis of Osteoclast Development

**DOI:** 10.3389/fcell.2021.657935

**Published:** 2021-07-13

**Authors:** Áron Pánczél, Simon P. Nagy, János Farkas, Zoltán Jakus, Dávid S. Győri, Attila Mócsai

**Affiliations:** Department of Physiology, Semmelweis University School of Medicine, Budapest, Hungary

**Keywords:** osteoclasts, cell fusion, fluorescence microscopy, Cre recombinase, myeloid cell differentiation, fluorescent Cre-reporter

## Abstract

Osteoclasts are multinucleated cells of hematopoietic origin which are critically involved in physiological and pathological bone resorption. They develop from myeloid progenitors through characteristic gene expression changes and intercellular fusion. This process is directed by M-CSF and RANKL which are also able to trigger osteoclast development from bone marrow cells *in vitro*. Osteoclasts are conventionally visualized by histochemical staining followed by manual counting, which hinders kinetic studies and automated quantification. Here we describe two fluorescence-based assays for the real-time analysis of myeloid cell to osteoclast development (FRAMCO) in primary mouse bone marrow cell cultures. Both assays rely on red-to-green fluorescence conversion of the membrane-targeted tdTomato/membrane-targeted eGFP (mTmG) transgene by Cre recombinase driven by the osteoclast-specific cathepsin K promoter (Ctsk-Cre). In the first assay (FRAMCO1.1), osteoclast-specific gene expression triggers red-to-green color conversion of cells carrying both the Ctsk-Cre and mTmG transgenes. In the second assay (FRAMCO1.2), red-to-green fluorescence conversion is triggered by fusion of neighboring co-cultured bone marrow cells separately carrying either the Ctsk-Cre or the mTmG transgenes. The two assays were tested using a high-content confocal fluorescence imaging system, followed by automated quantification. The FRAMCO1.1 assay showed robust red-to-green fluorescence conversion of more than 50% of the culture (including mononuclear cells) within 3 days under osteoclastogenic conditions. The FRAMCO1.2 assay showed a less robust but still readily measurable red-to-green color conversion in multinuclear cells within 5 days of differentiation. The assays required both the Ctsk-Cre and the mTmG transgenes and gave no signals in parallel macrophage cultures. The proper functioning of the two assays was also confirmed at the DNA, mRNA and bulk protein level. The assay systems were validated using lisophosphatidylcholine, a previously reported inhibitor of preosteoclast fusion. Taken together, our assays allow high-throughput automated real-time analysis of two critical aspects of osteoclast development, facilitating the screening for novel drug candidates for the pharmacological control of osteoclast-mediated bone resorption.

## Introduction

Osteoclasts are multinucleated cells uniquely capable of degrading bone tissue (Teitelbaum, 2000). They are critically involved in bone resorption required to maintain skeletal homeostasis, as indicated by the increased bone mass in osteopetrosis, a disease caused by inherited defects of osteoclast development or function (Sobacchi et al., 2013). Besides their role in normal bone turnover, osteoclasts also play a critical role in pathological bone loss in diseases, such as osteoporosis, rheumatoid arthritis, and osteolytic bone metastases (Rachner et al., 2011; Kitazawa et al., 2018; Shim et al., 2018; Győri and Mócsai, 2020). The role of osteoclasts in these diseases is also indicated by the therapeutic efficacy of anti-osteoclast therapeutics, such as bisphosphonates, RANKL-inhibiting monoclonal antibodies, or cathepsin K inhibitors (Russell, 2011; Lacey et al., 2012; Duong le et al., 2016).

Osteoclasts develop from the myeloid lineage of the hematopoietic system [either from yolk-sack derived erythromyeloid or bone marrow-derived myeloid progenitors (Jacome-Galarza et al., 2019; Yahara et al., 2020)] and share several characteristics with certain macrophage subsets. The development of osteoclast progenitors into mature osteoclasts is primarily directed by two cytokines, M-CSF, which promotes myeloid cell proliferation and differentiation, and RANKL, which triggers an osteoclast-specific differentiation program. Administration of M-CSF and RANKL to bone marrow cells is able to induce development of mature osteoclast-like cells even in *in vitro* cell culture.

Osteoclast development is characterized by two conceptually different aspects of differentiation: osteoclast-specific gene expression changes and intercellular fusion. During osteoclast-specific transcriptional activity, the primarily osteocyte/osteoblast-derived (Nakashima et al., 2011) RANKL cytokine triggers dramatic upregulation of a number of genes, such as *Acp5* (encoding tartrate-resistant acidic phosphatase or TRAP), *Ctsk* (cathepsin K), or *Nfatc1* (encoding NFATc1, the master regulator of osteoclast development), giving rise to mostly mononuclear preosteoclasts. During intercellular fusion, preosteoclasts fuse to each other to form large multinuclear syncytia (osteoclasts) which are able to spread over and resorb the underlying bone tissue. Though osteoclast-specific gene expression is believed to mostly precede preosteoclast fusion, there is substantial temporal overlap between the two processes.

**FIGURE 1:**
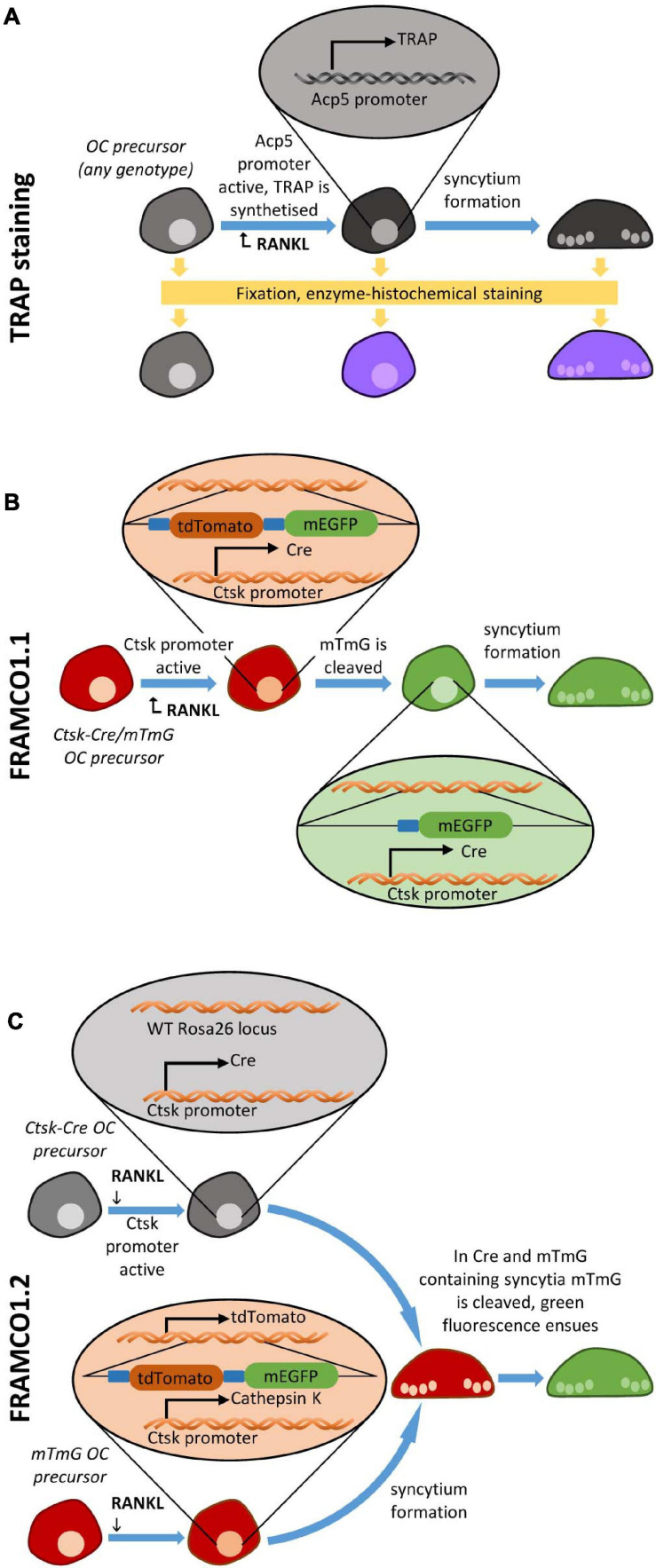
Overview of osteoclastogenesis assays. Schematic depiction of **(A)** the TRAP staining-based osteoclast detection, **(B)** the fluorescent Ctsk-Cre/mTmG-based (FRAMCO1.1) Ctsk-reporter system for the analysis of osteoclast-specific gene expression, and **(C)** the Ctsk-Cre + mTmG co-culture based (FRAMCO 1.2) fluorescent intercellular fusion assay.

The standard approach to visualize osteoclasts *in vitro* or *in vivo* is based on the histochemical staining for the TRAP enzyme (Minkin, 1982) and the presence of multiple nuclei in a single cell (Figure 1A). In this case, TRAP-positivity correlates with osteoclast-specific gene expression, whereas multinucleation is an indicator of intercellular fusion. However, analysis of osteoclast development based on TRAP staining has a number of shortcomings. Most importantly, the cells need to be fixed prior to staining, therefore it is not possible to follow the same cell culture over a prolonged period of time. In addition, counting TRAP-positive multinuclear cells is a very time-consuming process that is rather difficult to automate.

There has been a number of attempts to overcome the above limitations. Transgenic expression of the red fluorescent tdTomato protein under the control of the *Acp5* promoter allowed the fluorescence-based real-time analysis of TRAP expression as a measure of osteoclast-related gene expression (Kikuta et al., 2013). There have also been several attempts to visualize preosteoclast fusion, based on the analysis of the co-culture of two cell sources labeled by two different chemical stains or expressing two different fluorescent proteins (Levaot et al., 2015; Verma et al., 2018). However, the resources for the live imaging of osteoclast cultures are still rather limited. In addition, some aspects of the above approaches, such as the lack of internal reference for overall cell number or the difficulties of separating accidentally overlapping fluorescence signals from true fusion events, pose significant technical challenges.

The above issues prompted us to develop two separate fluorescence-based assays for the real-time analysis of osteoclast-specific gene expression (FRAMCO1.1) and intercellular fusion of preosteoclasts (FRAMCO1.2). Both assays rely on the conversion of red to green fluorescence by Cre-mediated recombination in *in vitro* osteoclast cultures. This is triggered by *Ctsk* (cathepsin K) expression (FRAMCO1.1) and intercellular fusion (FRAMCO1.2), whereas red fluorescence serves as internal reference for overall cell density. We also provide further non-fluorescence-based evidence of corresponding changes at the DNA, mRNA and protein level. Together with a related recent paper (Li et al., 2018), our experiments may contribute to the improved methodologies of studying osteoclast development in the future.

## Materials and Methods

### Animals

Mice carrying the *Gt(ROSA)26Sor^TM 4(*ACTB*–*tdTomato,–EGFP)Luo*^* (referred to as *Rosa26*^*mTmG*^ or mTmG) knock-in mutation (Muzumdar et al., 2007) were obtained from the Jackson Laboratory and were maintained in homozygous (*Rosa26*^*mTmG/mTmG*^) form. Mice carrying the *Ctsk^TM 1(*cre)Ska*^* (referred to as *Ctsk*^*Cre*^ or Ctsk-Cre) knock-in allele (Nakamura et al., 2007) were obtained from Shigeaki Kato (University of Tokyo) and were maintained in heterozygous (*Ctsk*^*Cre/*+^) form. The two strains were crossed to obtain *Ctsk*^*Cre/*+^*Rosa26*^*mTmG/mTmG*^ (referred to as Ctsk-Cre/mTmG) mice. All mice were on the C57BL/6 genetic background. Where indicated, wild-type C57BL/6 mice from our colony (originally obtained from the Jackson Laboratory) were used as controls (referred to as WT). Mice of both sexes were used. The vast majority of animals were between 10 and 16 weeks of age. No obvious difference between sexes or age groups were observed. Animals were kept in individually ventilated cages in a specific pathogen-free facility. All experiments were approved by the Animal Experimentation Review Board of Semmelweis University.

### *In vitro* Osteoclast Cultures

Osteoclasts were generated as previously described (Kertész et al., 2012; Győri et al., 2014; Csete et al., 2019). Long bones from hind limbs of adult mice of the indicated genotypes were dissected, cut open at proximal and distal metaphyses and flushed with sterile PBS. Following erythrocyte lysis with ammonium-chloride-potassium buffer, cells were pelleted and resuspended in α-MEM (Sigma) supplemented with 10% fetal bovine serum (FBS; Euroclone), 2 mM L-glutamine, 10 mM HEPES (pH ≈ 7.3), 100 U/ml penicillin and 100 μg/ml streptomycin (all from Sigma) and cultured in the presence of 10 ng/ml recombinant mouse M-CSF (PeproTech) in a standard CO_2_ incubator. After two days (referred to as Day 0), non-adherent cells were harvested, diluted to a final density of 10^5^ cells/cm^2^, supplemented with 50 ng/ml M-CSF only for macrophage, or with 50 ng/ml M-CSF and 50 ng/ml RANKL (PeproTech) for osteoclast cultures, and placed on tissue culture plates (Greiner Bio-One μClear^®^ Cellstar^®^ 655090 for confocal microscopy and Corning Costar 3516 for non-fluorescence-based studies). The cultures were then followed for up to 6 days with the culture medium replaced every 2 days. For representative images, cultures were fixed and stained for tartrate resistant acid phosphatase using the Acid Phosphatase, Leukocyte (TRAP) kit (387A, Sigma). For inhibitor studies, 12:0 lysophosphatidylcholine (1-lauroyl-2-hydroxy-sn-glycero-3-phosphocholine, referred to as LPC) was obtained from Avanti^®^, dissolved in sterile water and added to the cultures at the indicated final concentrations.

### Fluorescence Microscopy

Cell cultures were imaged with an ImageXpress Micro Confocal High Content Imaging System (Molecular Devices). Filter pairs of 377/50 and 447/60 nm, 474/27 and 525/45 nm or 562/40 and 624/40 nm were used for blue, green and red fluorescence, respectively. For representative images and nucleus-based quantification, a nuclear staining with 0.5 μg/ml Hoechst 33342 dye (Invitrogen) in PBS was applied for 20 min.

For quantitative kinetic analysis, cultures were imaged immediately after plating, then kept in a standard CO_2_ incubator and taken to the microscope for further imaging at room temperature and atmospheric CO_2_ concentration at the indicated time-points. Imaging of one 96-well plate took approximately 40 min which the cells seemed to tolerate reasonably well. During image acquisition, six 10× magnified fields of view (each covering 1.96 mm^2^) were captured from each well and at least four parallel wells were imaged for each condition. Images were quantified with MetaXpress High Content Image Acquisition & Analysis Software to determine the area of objects of interest based on intensity above local background as well as minimal and maximal object sizes. The segmentation workflow used did not attempt to separate touching/overlapping suprathreshold objects, thus only the total area and not the object count was used for further quantitative characterization of the cultures. Detection and quantification of nuclei was likewise performed in the MetaXpress software using a built-in algorithm that determined both the total number of nuclei and the number of nuclei within the objects identified by segmentation of the green channel images.

For real-time time-lapse video microscopy, cell cultures were incubated in a standard CO_2_ incubator for 48 or 60 h after RANKL addition, then the medium was exchanged, and the culture plate was placed in the ImageXpress system with a sealing ring on top to create a closed space around the wells where a 5% CO_2_–95% air gas mixture, humidity and a constant temperature of 37°C was provided for the cells using the ImageXpress system’s built-in incubation capabilities. Images were taken every 60 min. Videos were processed using ImageJ/Fiji, which involved correction for background bleaching throughout the time-series using the BaSiC plugin (Peng et al., 2017).

### Genomic PCR Analysis

DNA from cultured cells was isolated using an SDS, Tris, EDTA (all from Sigma) and proteinase K (Qiagen) based lysis followed by purification using phenol-chloroform-isoamyl alcohol (Sigma). Cre-mediated recombination was followed by the 5′-CGTGCTGGTTATTGTGCTGTCTC-3′ forward and 5′-CCATTCTCCTGTCCGTTCGCTT-3′ reverse primers yielding a 261 bp product from the recombined (mG) version of the mTmG construct. It should be noted that the design of the mTmG construct does not allow for specific amplification of the recombined allele since any primer binding sites would also be present in the intact mTmG allele. However, the larger (2,674 bp) product amplified from the intact mTmG allele is not expected to interfere with the identification of the mG allele and is therefore not shown in the figures. A different pair of 5′-AATGGGAGCAGTGGTGGAAT-3′ forward and 5′-ATGTTGAGAGTCAGCAGTAGCC-3′ reverse primers was used to amplify a 98 bp segment in the loxP-flanked, tdTomato-encoding portion of the mTmG construct. For loading control, a part of the Rosa26 locus unaffected by the knock-in genetic modification, extending over 689 bp, was amplified using the 5′-GAACACCACCTGACGGGAGA-3′ forward and 5′-AGATGGGCGGGAGTCTTCTG-3′ reverse primers. Reaction products were run on 2% agarose gels and imaged on a Bio-Rad^TM^ Gel Doc XR+ system.

### RT-qPCR Assay

Total RNA was isolated from cultures using the NucleoSpin RNA kit (Macherey-Nagel) and the RNeasy Plus Mini Kit (Qiagen) according to the manufacturer’s instructions. Total RNA concentration was determined by a NanoDrop spectrophotometer and cDNA synthesis was performed using the QuantiTect and QuantiNova Reverse Transcription Kits (Qiagen). Reactions were purified using ammonium–acetate-based ethanol precipitation and resuspended in Tris-EDTA buffer. FAM conjugated TaqMan Gene Expression Assays for *Ctsk* (Mm00484039_m1), *Acp5* (Mm00475698_m1), *Nfatc*1 (Mm00479445_m1), *Cre* (custom assay #APRWJMG designed with Thermo Fisher’s web application), *Egfp* (Mr04097229_mr), and endogenous control *Gapdh* (Mm99999915_g1) were obtained from Thermo Fisher Scientific. Quantitative PCR was performed using LightCycler 480^®^ Probes Master (Roche) on a Roche LightCycler 480^®^ II instrument. For quantification, the standard-curve based Efficiency Method was applied.

### Immunoblotting

Immunoblotting was performed essentially as described (Győri et al., 2014; Csete et al., 2019). Cells grown on 6-well tissue culture plates were washed with ice-cold PBS and lysed using radioimmunoprecipitation assay buffer (RIPA, containing 1% Triton X, 0.1% SDS, 0.5% sodium deoxycholate, 30 mM HEPES, 5 mM Na-EGTA, 10 mM benzamidine, and 20 mM sodium fluoride in physiological saline) supplemented with sodium-orthovanadate, phosphatase inhibitor cocktails 1 and 2, PMSF and aprotinin (all from Sigma). Cell debris was removed by centrifugation at 16,100 g. Protein concentration was determined using the Micro BCA^TM^ Protein Assay Kit (Thermo Fisher Scientific). Samples then were mixed with 4× reducing sample buffer and boiled for 10 min. Ten micrograms of total protein was run on a 14% SDS-polyacrylamide gel, electroblotted onto nitrocellulose membranes and stained with Ponceau. Membranes were then blocked with 3% dry milk in PBS and 0.1% Tween 20 (PBS-Tween), followed by immunoblotting with primary antibodies against eGFP (1:2,000; clone F56-6A1.2.3; Abcam) or β-actin (1:10,000; Clone AC-74; Sigma) diluted in 3% BSA in PBS-Tween, followed by peroxidase-labeled anti-mouse IgG antibodies (1:5,000; GE Healthcare) diluted in 3% dry milk in PBS-Tween. Signal was developed by ECL (GE Healthcare) and exposed to X-ray film.

### Presentation of the Data

Experiments were performed the indicated number of times. Quantitative results show mean and SEM from all independent experiments performed.

## Results

### Overview of the Osteoclast-Specific Gene Expression Assay

Our first aim was to develop an assay for the fluorescence-based real-time analysis of osteoclast-specific changes of gene expression. To this end, we crossed mice carrying the *Ctsk*^*Cre*^ (referred to as Ctsk-Cre) knock-in mutation allowing the expression of the Cre recombinase from the endogenous *Ctsk* locus in an osteoclast-specific manner (Nakamura et al., 2007) with mice carrying the *Rosa26*^*mTmG*^ (referred to as mTmG) mutation which leads to the ubiquitous expression of the red fluorescent membrane-targeted tdTomato (“mT”) protein but allows switching to ubiquitous expression of the green fluorescent membrane-targeted eGFP (“mG”) upon Cre-mediated recombination (Muzumdar et al., 2007). As shown in the schematic representation in Figure 1B, presence of both mutations in the same cells (referred to as Ctsk-Cre/mTmG cells) can be considered a Ctsk reporter system which is expected to result in red fluorescence under basal conditions but conversion to green fluorescence upon activation of the *Ctsk* gene during osteoclast differentiation. We have designated this system as FRAMCO1.1, standing for fluorescence-based real-time analysis of myeloid cell to osteoclast differentiation, version 1.1.

### Emergence of Green Fluorescence in Osteoclasts but Not Macrophages

**FIGURE 2:**
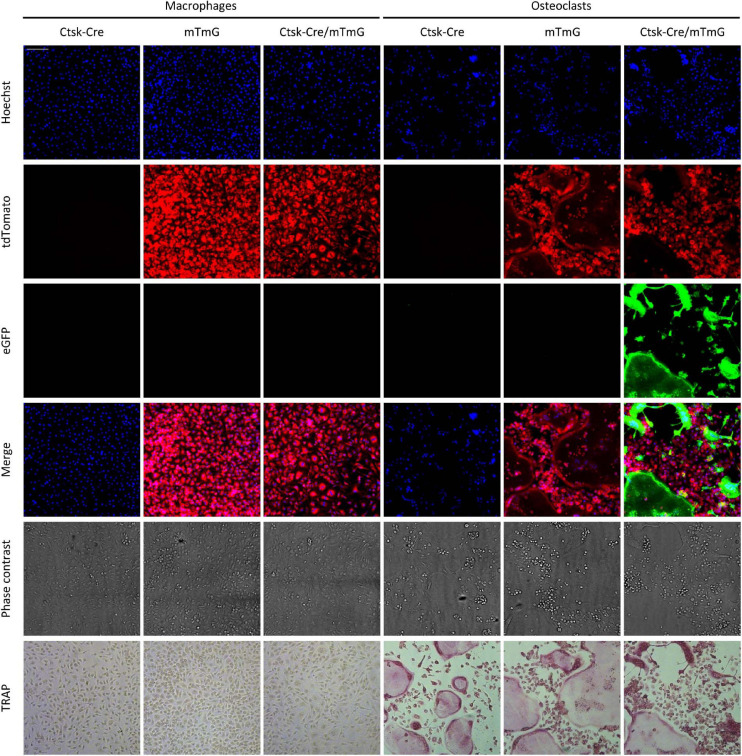
Fluorescence and brightfield images of the FRAMCO1.1 assay. Representative images of macrophage and osteoclast cultures taken on Day 5 of differentiation (where Day 0 is defined as the time RANKL is first added to osteoclast cultures). Nuclei were stained with Hoechst dye. The first three rows show images from the blue, red and green fluorescence channels, supposedly corresponding to signals from the indicated fluorophores. The cells were fixed and stained for TRAP after taking fluorescence and phase contrast images. Each column shows images from the same field of view. The scale bar corresponds to 100 μm and applies to all images. Images are representative of 8–10 independent experiments.

The above approach was tested on *in vitro* cultures of mouse bone marrow-derived myeloid progenitors differentiated toward macrophages in the presence of 50 ng/ml M-CSF or toward osteoclasts in the presence of 50 ng/ml M-CSF and 50 ng/ml RANKL. The cells were cultured for 5 days, stained with Hoechst 33342 dye to label the nuclei, and then imaged using an ImageXpress confocal high-content fluorescence imaging system (Figure 2). The cells were fixed and stained for TRAP immediately after capturing the fluorescence images. As shown in Figure 2, osteoclast cultures contained large multinucleated TRAP-positive cells (supposedly osteoclasts), whereas neither TRAP-positive nor multinuclear cells were present in macrophage cultures. Ctsk-Cre cells (without the mTmG mutation) showed no substantial red or green fluorescence irrespective of the culture conditions, although cells and nuclei were clearly present in the cultures. In contrast, mTmG cells (in the absence of the Ctsk-Cre mutation) showed a constitutive red fluorescence signal that did not differ between macrophage and osteoclast cultures (except for the different morphologies of the two cell types). Ctsk-Cre/mTmG macrophage cultures were similar to mTmG macrophages. No green fluorescence emerged in any of Ctsk-Cre or mTmG single mutant cultures or in Ctsk-Cre/mTmG macrophages. Importantly, however, a robust green fluorescence signal could be observed in Ctsk-Cre/mTmG osteoclast cultures. This green fluorescence mostly co-localized with large multinucleated TRAP-positive cells (osteoclasts), though apparently mononuclear green fluorescent cells (supposedly preosteoclasts) could also be observed. Most green fluorescent areas were largely devoid of red fluorescence, indicating a high efficiency of red-to-green color conversion. These snapshot images indicated that the Ctsk-Cre/mTmG mutation (FRAMCO1.1 assay) is capable of identifying cells that have undergone upregulation of osteoclast-specific genes.

### Time-Course of the Appearance of Green Fluorescence

**FIGURE 3:**
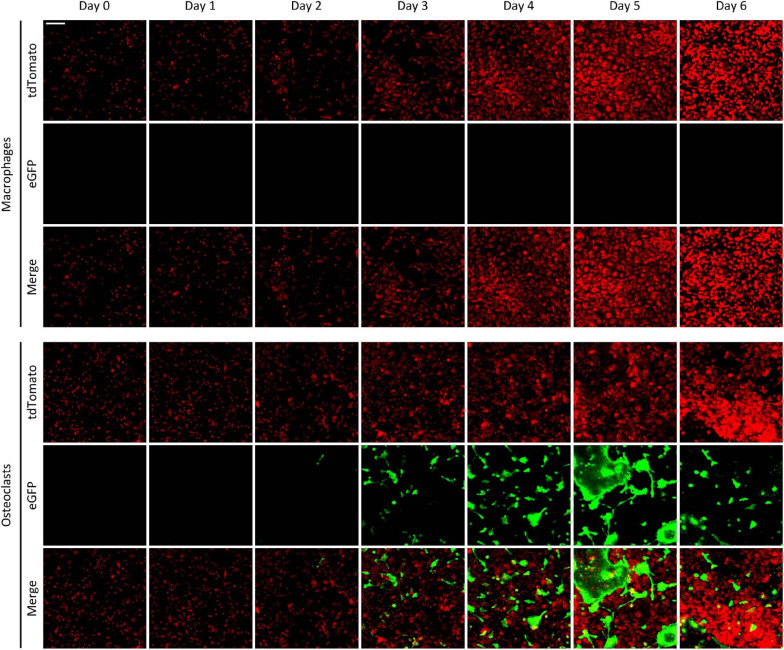
The time-course of fluorescence changes in the FRAMCO1.1 assay. Representative images of Ctsk-Cre/mTmG macrophages and osteoclasts captured on the indicated days (Day 0 is the time RANKL was first added to osteoclast cultures). Red and green channels supposedly correspond to the indicated fluorophores. The scale bar corresponds to 100 μm and applies to all images. Images are representative of 8–10 independent experiments.

We have also tested the time course of the emergence of green fluorescence signals in the Ctsk-Cre/mTmG cultures, exploiting the possibility of repeated imaging of the same cultures and the same fields of view. As shown in Figure 3, only red but not green fluorescence was observed in Ctsk-Cre/mTmG macrophage cultures. In contrast, a substantial number of green fluorescent cells emerged from Day 3 in osteoclast cultures, indicating activation of the osteoclast-specific *Ctsk* gene. These green fluorescent cells became larger by Day 5, supposedly indicating development of large multinucleated osteoclasts. However, both the number and size of green cells were reduced by Day 6, likely reflecting apoptosis of large multinucleated cells. Again, most of the green fluorescent cells showed strongly reduced red fluorescence, indicating effective red-to-green color conversion. We have also generated videos of Ctsk-Cre/mTmG macrophage and osteoclast cultures with higher temporal resolution between Days 2–5 after RANKL addition. One such video is shown as Supplementary Video 1 to demonstrate the time-lapse capabilities of our FRAMCO1.1 system. Taken together, our Ctsk-Cre/mTmG (FRAMCO1.1) approach allows the fluorescence-based kinetic analysis of osteoclast-specific gene expression during the differentiation of myeloid progenitors toward the osteoclast lineage.

### Quantitative Analysis of the Fluorescence Signals

One of the major advantages of our system is that the emergence of green fluorescence can be readily quantified over a prolonged time during long-term imaging of the same osteoclast cultures. In addition, even eGFP-negative cells can be conveniently detected based on their red fluorescence, allowing the normalization for the total cellular content and therefore the exclusion of effects (e.g., differential proliferation) that are independent of red-to-green color conversion.

**FIGURE 4:**
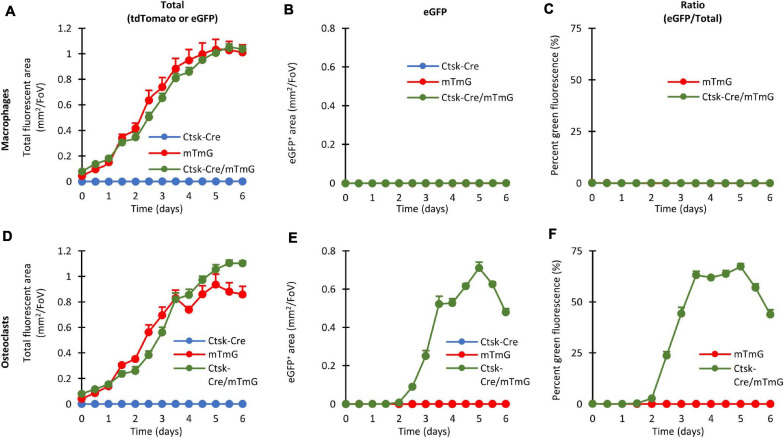
Quantitative evaluation of the fluorescent signals in the FRAMCO1.1 assay. Quantification of the fluorescence signals of macrophage **(A–C)** or osteoclast **(D–F)** cultures of the indicated genotypes at the indicated time points. Graphs depict the kinetics of the total (tdTomato, eGFP or both) fluorescent area **(A,D)**, green (eGFP-positive) fluorescent area **(B,E)** and the ratio of the two areas **(C,F)**. All panels depict mean and SEM from 5 to 10 independent experiments.

Results of the quantification of such experiments are shown in Figure 4. Panels A–C show results of macrophage cultures, whereas Panels D–F depict results from osteoclast cultures. All panels derive from analysis of fluorescent objects identified using built-in ImageXpress algorithms which quantify the area of objects showing red or green fluorescence above local background. Figures 4A,D show the area of all (red or green, i.e., tdTomato- or eGFP-expressing) fluorescent objects, as an approximation of total cellular area. Figures 4B,E show the area of green fluorescent (eGFP-expressing) objects, i.e., where red-to-green color conversion due to activation of the *Ctsk* gene has occurred. The percentage of the area of green fluorescent (eGFP) objects among all fluorescent (tdTomato or eGFP) ones, reflecting the relative extent of red-to-green color conversion, is depicted in Figures 4C,F. Besides the Ctsk-Cre/mTmG double-mutant osteoclast cultures, Ctsk-Cre and mTmG single-mutant cultures and parallel macrophage cultures are also shown as reference.

Likely reflecting the proliferation of the cells, the area of all (tdTomato or eGFP) fluorescence gradually increased in Ctsk-Cre/mTmG macrophage cultures (Figure 4A), whereas practically no eGFP-positive cells could be detected (Figure 4B). Therefore, in agreement with the lack of osteoclast-specific gene expression, the percentage of the area of green fluorescent objects remained practically zero in Ctsk-Cre/mTmG macrophage cultures (Figure 4C). Similar changes were also seen in mTmG macrophage cultures, whereas no fluorescence was observed in Ctsk-Cre cultures (Figures 4A–C).

The overall level of red or green (tdTomato or eGFP) fluorescence also gradually increased in mTmG and Ctsk-Cre/mTmG osteoclast cultures (Figure 4D). Importantly, however, a robust green fluorescence (eGFP) signal was observed in Ctsk-Cre/mTmG osteoclast cultures, whereas no such signals were seen in mTmG ones (Figure 4E). As a result, a high percentage of the area of fluorescent objects showed green fluorescence in Ctsk-Cre/mTmG but not in mTmG osteoclast cultures (Figure 4F). Further analysis of the Ctsk-Cre/mTmG curve in Figure 4F revealed that substantial eGFP expression arose by Day 3 and eGFP-positive area reached its maximum on Day 5 at >50% of the total fluorescent area, followed by a partial decline despite further apparent proliferation of the cells (Figure 4D). No fluorescence signal could be observed under any conditions in Ctsk-Cre osteoclast cultures (Figures 4D–F).

Taken together, the experiments shown in Figure 4 indicate that our fluorescence-based Ctsk reporter assay (FRAMCO1.1) is suitable for the long-term follow-up and quantification of osteoclast-specific gene expression. Analysis of control samples, such as single (Ctsk-Cre or mTmG) mutants or macrophages confirm the working principles and specificity of the assay, and the use of red fluorescence readouts provides internal reference for normalization. The >50% color conversion rate also indicates the robustness of our assay.

### Molecular Analysis of Cre-Mediated Recombination and eGFP Expression

Besides fluorescence-based analyses, we also wanted to perform bulk population-based assays on the effects of *Ctsk* promoter-driven Cre expression at the DNA, mRNA, and protein level.

**FIGURE 5:**
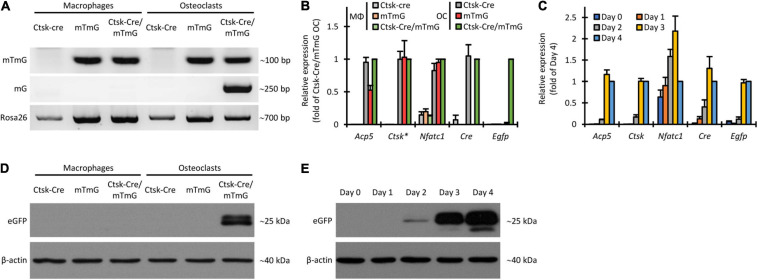
Non-fluorescence-based assessment of Cre-mediated recombination in the FRAMCO1.1 assay. **(A)** Genomic PCR analysis of the indicated cultures on Day 4 using primers amplifying a target in the loxP-flanked portion of the intact mTmG construct (upper row), primers yielding an approximately 250 bp product from the Cre-recombined (mG) allele (middle row) or with primers amplifying a portion of the *Rosa26* locus downstream of the mTmG insertion site (lower row). **(B)** RT-qPCR analysis of different transcripts on Day 4 in macrophage (MΦ) or osteoclast (OC) cultures. The asterisk indicates that *Ctsk* expression values for samples carrying the Ctsk-Cre mutation were multiplied by 2 to compensate for the haploinsufficiency of the wild-type *Ctsk* allele. All samples were normalized to Ctsk-Cre/mTmG osteoclast values. **(C)** RT-qPCR analysis of the time course of expression of the indicated genes during differentiation of Ctsk-Cre/mTmG osteoclasts. Data were normalized to Day 4 expression of the respective markers. **(D,E)** Immunoblot experiments detecting eGFP or β-actin as a loading control from whole-cell lysates of the indicated cultures on Day 4 **(D)** or of Ctsk-Cre/mTmG osteoclast cultures at the indicated time points **(E)**. Panels **(A,D,E)** are representative of 3–5 independent experiments. Panels **(B,C)** show mean and SEM from 4 to 7 independent experiments.

We first designed and utilized allele-specific PCR protocols to distinguish between the intact mTmG transgene and its truncated mG version, generated by Cre-mediated excision of the mT segment. Results of such experiments on genomic DNA are shown in Figure 5A. As expected, the intact (mTmG) allele was absent from Ctsk-Cre cultures (without mTmG) but it was readily detected in all mTmG or Ctsk-Cre/mTmG macrophage and osteoclast cultures. In contrast, the mG allele was only present in Ctsk-Cre/mTmG osteoclast cultures but not in other osteoclast or any macrophage samples, confirming the genetic principle of the FRAMCO1.1 system and indicating successful Cre-mediated excision of the mT part of the mTmG transgene only in osteoclasts but not in macrophages. As a reference, we have also performed a PCR assay to detect a downstream Rosa26 sequence. This PCR protocol recognized all samples, although the amplification of the Ctsk-Cre samples was somewhat less robust (the reason for this difference is at present unclear).

Next we performed quantitative RT-PCR-based analysis of the expression of the osteoclast-related *Acp5*, *Ctsk*, and *Nfatc1* genes, as well as of the *Cre* transgene and the *Egfp* reporter. As shown in Figure 5B, *Acp5*, *Ctsk*, and *Nfatc1* expression showed robust upregulation in all osteoclast samples compared to parallel macrophage cultures by Day 4. *Cre* was expressed in Ctsk-Cre and Ctsk-Cre/mTmG but not in mTmG osteoclasts, nor in any macrophage cultures. Importantly, *Egfp* was only expressed in Ctsk-Cre/mTmG osteoclast cultures (Figure 5B), indicating the strict control of eGFP expression. Further kinetic analysis in Ctsk-Cre/mTmG osteoclast cultures (Figure 5C) also revealed substantial upregulation of *Acp5*, *Ctsk*, and *Nfatc1* expression during osteoclastic differentiation. As expected, *Cre* expression mostly followed that of *Ctsk*, and *Egfp* expression showed only a very modest delay compared to *Ctsk* expression.

We have also tested the appearance of the eGFP protein by immunoblotting. As shown in Figure 5D, eGFP was present in Ctsk-Cre/mTmG osteoclast cultures but not in osteoclast samples carrying only one of the two mutations, or in any of the macrophage cultures. Kinetic analysis of eGFP protein levels in Ctsk-Cre/mTmG osteoclast cultures (Figure 5E) showed the first appearance of eGFP as a faint band on Day 2 and robust eGFP signals on Days 3 and 4. This was in line with the appearance of *Egfp* mRNA (Figure 5C).

Taken together, we have been able to document the DNA-level cleavage of the mTmG transgene, as well as the expression of eGFP at the mRNA and protein level in the FRAMCO1.1 system. All these responses required the simultaneous presence of the Ctsk-Cre and mTmG mutations, and were under the strict control of RANKL-induced osteoclastic differentiation, confirming both the genetic concept and the osteoclast-specific nature of the FRAMCO1.1 assay.

### Overview of the Preosteoclast Fusion Assay

After having established a robust fluorescence-based assay for osteoclast-specific gene expression changes, our next aim was to set up another fluorescence-based assay for the analysis of the fusion of preosteoclasts to large multinucleated cells. We used the same Ctsk-Cre and mTmG mutations for this assay, but in a substantially different manner (Figure 1C). The principle of our fusion assay was the co-culture of Ctsk-Cre and mTmG single-mutant cells (referred to as “Ctsk-Cre + mTmG” cultures), where no cells carried both the Ctsk-Cre and mTmG mutations at the beginning of the culture period. Since Cre and its mTmG substrate would be present in different (although possibly neighboring) cells, Cre would not have access to its target and therefore no mTmG cleavage and no eGFP expression would occur. Subjecting these cells to osteoclastogenic conditions would be expected to initiate the fusion of neighboring Ctsk-Cre and mTmG cells and the cleavage of the mTmG transgene to its truncated mG variant, resulting in eGFP expression and emergence of green fluorescence. Therefore, red-to-green fluorescent color conversion in this assay would be the result of the intercellular fusion process. We have designated this system as FRAMCO1.2, standing for fluorescence-based real-time analysis of myeloid cell to osteoclast differentiation, version 1.2.

### Appearance of Green Fluorescence Upon Preosteoclast Fusion

**FIGURE 6:**
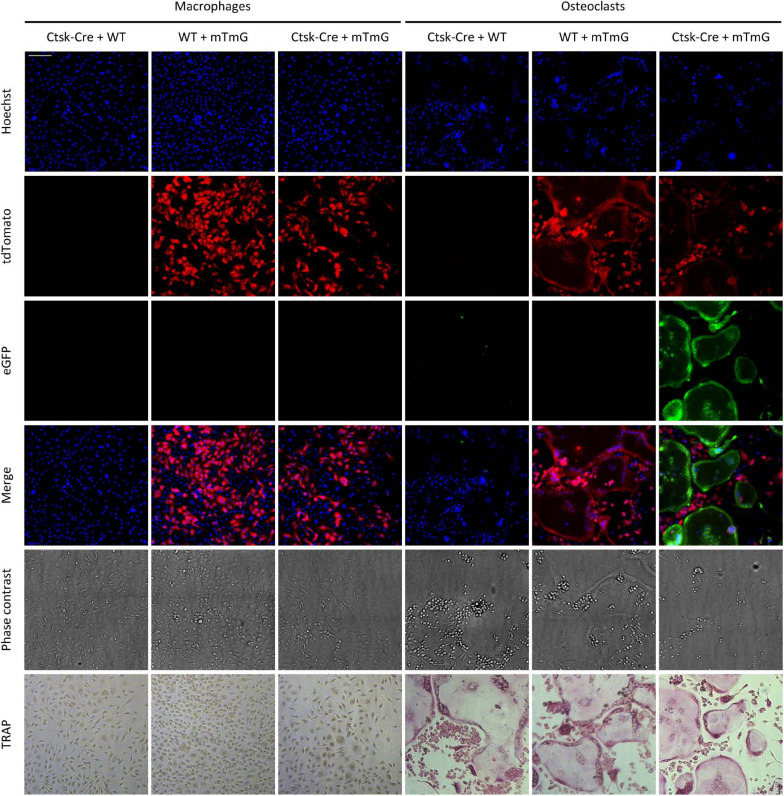
Fluorescence and brightfield images of the FRAMCO1.2 (intercellular fusion) assay. Representative images of macrophage and osteoclast co-cultures on Day 5 after RANKL addition. Nuclei were labeled with Hoechst. The first three rows show images from the blue, red, and green fluorescence channels, supposedly corresponding to signals from the indicated fluorophores. The cells were fixed and stained for TRAP after fluorescence imaging. Images in each column show the same field of view. The scale bar corresponds to 100 μm and applies to every image. Images are representative of 8–10 independent experiments.

To test the feasibility of the above approach, we mixed bone marrow-derived myeloid progenitors of two different genotypes at a 1:1 ratio and differentiated them toward macrophages or osteoclasts in the presence of 50 ng/ml M-CSF without or with 50 ng/ml RANKL, respectively. Representative fluorescence and phase-contrast images obtained on Day 5 after staining the nuclei with Hoechst 33342 dye, along with concomitant TRAP-stained images of the same fields of view from the same samples are shown in Figure 6. TRAP-stained images showed typical morphology of both macrophage (elongated TRAP-negative mononuclear cells) and osteoclast (large multinuclear TRAP-positive cells) cultures. Co-cultures of Ctsk-Cre and wild-type cells (referred to as “Ctsk-Cre + WT” cultures) did not show any red or green fluorescence. Co-cultures of wild-type and mTmG cells (referred to as “WT + mTmG” cultures) resulted in the appearance of a large number of red (tdTomato) but no green (eGFP) fluorescent cells. In line with the expected lack of intercellular fusion, only red but no green fluorescence could be observed in Ctsk-Cre + mTmG macrophage co-cultures. However, a robust green (eGFP) fluorescence signal emerged in Ctsk-Cre + mTmG co-cultures differentiated toward osteoclasts. This green fluorescence was practically entirely localized to large multinucleated osteoclast-like cells, whereas mononuclear cells remained non-fluorescent (supposedly Ctsk-Cre cells) or showed only red fluorescence (supposedly mTmG cells). Green cells also showed reduced red fluorescence, indicating relatively efficient red-to-green color conversion. This pattern suggests that the emergence of green fluorescence indeed resulted from the fusion of neighboring Ctsk-Cre and mTmG cells, allowing Cre to access its mTmG target, as expected from the original design of the FRAMCO1.2 system.

### Time-Course of Fusion-Induced Fluorescence

**FIGURE 7:**
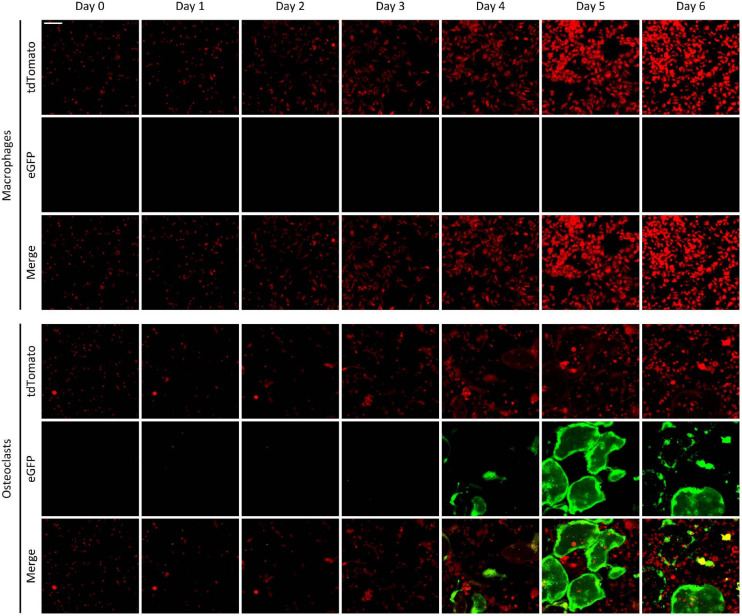
The time-course of fluorescence changes in the FRAMCO1.2 assay. Representative images of Ctsk-Cre + mTmG macrophage and osteoclast co-cultures captured on the indicated days after RANKL addition. Red and green channels supposedly correspond to the indicated fluorophores. The scale bar corresponds to 100 μm and applies to all images. Images are representative of 8–10 independent experiments.

We also followed the same cultures over a prolonged period of time. Representative images of Ctsk-Cre + mTmG co-cultures differentiated toward macrophages or osteoclasts are shown in Figure 7. As expected, no green (eGFP) fluorescence could be observed at any time points in macrophage cultures. On the other hand, green fluorescent cells started to emerge in Ctsk-Cre + mTmG osteoclast cultures around Day 4, primarily localized to relatively small but already likely multinuclear cells. Green (eGFP) fluorescence substantially increased by Day 5, primarily through the emergence of very large and spread-out green fluorescent cells, whereas green fluorescence declined again by Day 6, likely reflecting apoptosis of the large eGFP-positive cells. Similar to Figure 6, practically no green fluorescence was seen in supposedly mononuclear cells. We have also generated videos of Ctsk-Cre + mTmG osteoclast cultures with higher temporal resolution between Days 2–5 and Days 2.5–6 after RANKL addition. One video for each of the two timespans is shown as Supplementary Videos 2, 3 to demonstrate the time-lapse capabilities of our FRAMCO1.2 system. Taken together, these results suggest that the differentiation of Ctsk-Cre + mTmG co-cultures toward osteoclasts in our FRAMCO1.2 assay system is suitable for the kinetic analysis of the intercellular fusion process during osteoclast development.

### Quantification of Preosteoclast Fusion

**FIGURE 8:**
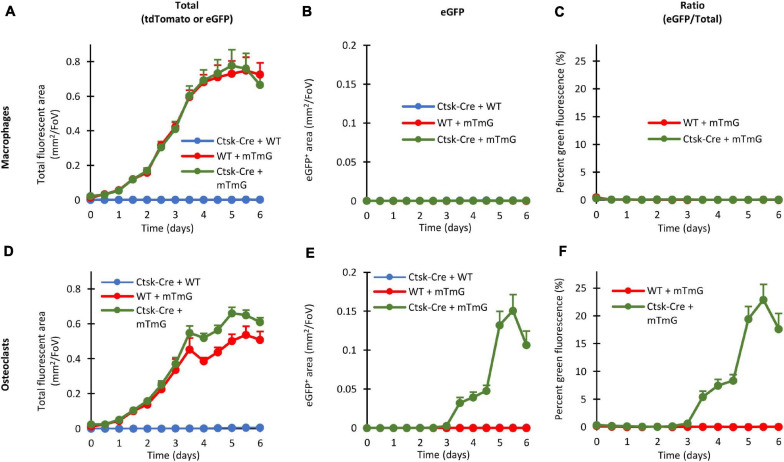
Quantitative evaluation of the fluorescent signals in the FRAMCO1.2 assay. Quantitative evaluation of macrophage **(A–C)** and osteoclast **(D–F)** co-cultures of the indicated genotypes either showing the changes in total (tdTomato + eGFP) fluorescent area **(A,D)**, in green (eGFP) fluorescent area **(B,E)** or in the green area as percentage of the total fluorescent area **(C,F)**. All charts depict mean and SEM from 5 to 10 independent experiments.

Quantification of fusion-induced eGFP expression is shown in Figure 8. As shown in Figure 8A, the area of all (red or green) fluorescent objects increased until Day 4 in Ctsk-Cre + mTmG or WT + mTmG macrophage co-cultures, whereas no fluorescence could be observed in Ctsk-Cre + WT macrophage co-cultures. As expected, practically no green (eGFP) fluorescence could be observed in any of the macrophage cultures (Figure 8B) and therefore the percentage green fluorescence (relative to total fluorescence) in Ctsk-Cre + mTmG and WT + mTmG macrophage co-cultures remained close to zero (Figure 8C). There was a steady increase in the total fluorescence until the end of the 6-day observation period in Ctsk-Cre + mTmG and WT + mTmG osteoclast co-cultures, whereas no fluorescence could be observed in Ctsk-Cre + WT samples (Figure 8D). Importantly, there was a well-measurable emergence of green (eGFP) fluorescence in Ctsk-Cre + mTmG but not in any other osteoclast co-cultures (Figure 8E). Accordingly, the percent green fluorescence (ratio of green and total fluorescence) showed a well-measurable increase in Ctsk-Cre + mTmG but not in WT + mTmG co-cultures differentiated toward osteoclasts (Figure 8F). This fluorescence signal showed a slower and then accelerated increase until its peak around Day 5, followed by a decline by Day 6 (likely reflecting apoptosis of osteoclasts). Since this signal required the presence of both Ctsk-Cre and mTmG cells and it was not present in macrophage cultures, it is reasonable to assume that this signal reflects the intercellular fusion between neighboring Ctsk-Cre and mTmG cells during osteoclast development, as expected from the original FRAMCO1.2 design.

Taken together, our Ctsk-Cre + mTmG co-culture-based assay (FRAMCO1.2) allows the quantitative fluorescence-based real-time analysis of intercellular fusion during osteoclast development.

### Non-fluorescent Assays to Test Intercellular Fusion

**FIGURE 9:**
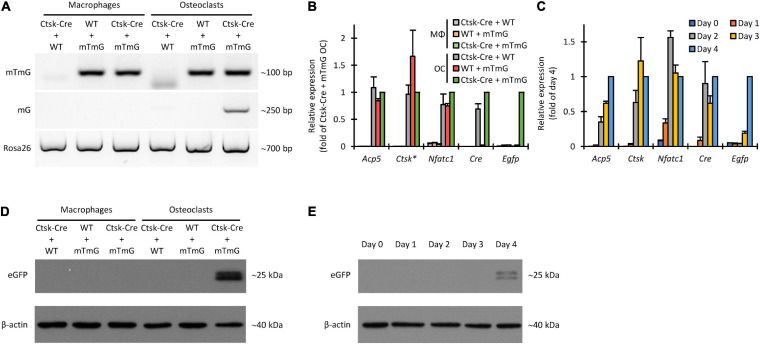
Non-fluorescence-based assessment of Cre-mediated recombination in the FRAMCO1.2 assay. **(A)** Genomic PCR analysis of Cre-mediated recombination with primers amplifying a loxP-flanked portion of the mTmG construct (upper row), an ~250 bp region from the recombined (mG) allele (middle row) or a downstream region of the *Rosa26* locus unaffected by the mTmG mutation (lower row). **(B)** RT-qPCR analysis of different transcripts on Day 4 in macrophage (MΦ) or osteoclast (OC) cultures. The asterisk indicates that *Ctsk* expression values for samples with half of the cell-population carrying the Ctsk-Cre mutation were multiplied by 4/3 to compensate for the haploinsufficiency of the wild-type *Ctsk* allele. All samples were normalized to Ctsk-Cre + mTmG osteoclast values. **(C)** RT-qPCR analysis of the time course of expression of the indicated genes during differentiation of Ctsk-Cre + mTmG osteoclast co-cultures relative to *Gapdh*. Data were normalized to Day 4 expression values of the respective transcripts. **(D,E)** Immunoblot experiments detecting eGFP and β-actin as loading control from whole cell lysates of the indicated co-cultures on Day 4 **(D)** or from Ctsk-Cre + mTmG osteoclast co-cultures on the indicated days **(E)**. Panels **(A,D,E)** are representative of 3–6 independent experiments. Panels **(B,C)** show mean and SEM from 4 to 7 independent experiments.

We also performed non-fluorescent assays to demonstrate the fusion-dependent cleavage of the mTmG transgene and the expression of eGFP. First, we tested the presence of the full (mTmG) and truncated (mG) versions of the mTmG transgene by allele-specific PCR from genomic DNA. As shown in Figure 9A, the intact mTmG allele could be readily amplified from samples where mTmG cells were present (together with wild-type or Ctsk-Cre cells), whereas no amplification occurred in Ctsk-Cre + WT co-cultures. Importantly, the PCR product corresponding to the truncated (mG) allele could only be amplified from Ctsk-Cre + mTmG osteoclast co-cultures, but not from any other one. It is reasonable to assume that this signal derived from Cre-mediated excision of the mT segment from the mTmG transgene as a result of fusion of Ctsk-Cre and mTmG preosteoclasts.

Next we tested expression of *Acp5*, *Ctsk*, *Nfatc1*, *Cre*, and *Egfp* at the mRNA level by quantitative RT-PCR. As shown in Figure 9B, *Acp5*, *Ctsk*, and *Nfatc1* were readily expressed in all osteoclast but not in parallel macrophage cultures by Day 4. Cre was expressed in Ctsk-Cre + WT and Ctsk-Cre + mTmG but not in WT + mTmG osteoclasts, nor in any macrophage cultures. Importantly, *Egfp* was only expressed in Ctsk-Cre + mTmG osteoclast cultures (Figure 9B), indicating the osteoclast-specificity of red-to-green color conversion. Further kinetic analysis in Ctsk-Cre + mTmG osteoclast cultures (Figure 9C) revealed substantial upregulation of *Acp5*, *Ctsk*, and *Nfatc1* expression during osteoclastic differentiation. As expected, *Cre* expression mostly reflected that of *Ctsk*, whereas *Egfp* expression showed a marked delay, possibly indicating a delay between *Ctsk* expression and completion of preosteoclast fusion.

We also tested the appearance of the eGFP protein by immunoblotting. As shown in Figure 9D, a measurable signal was seen in Ctsk-Cre + mTmG co-cultures differentiated toward osteoclasts, but not in any other samples. The time-course of this signal (Figure 9E) indicated that it began to emerge toward Days 3–4 of the osteoclast cultures. Again, this signal likely reflected fusion of Ctsk-Cre and mTmG cells under osteoclastogenic conditions.

Taken together, experiments testing mTmG cleavage and eGFP expression at the DNA, mRNA, and protein levels confirmed the suitability of the FRAMCO1.2 system to detect intercellular fusion in a strictly osteoclast-specific manner.

### Quantification Based on Nuclear Staining

**FIGURE 10:**
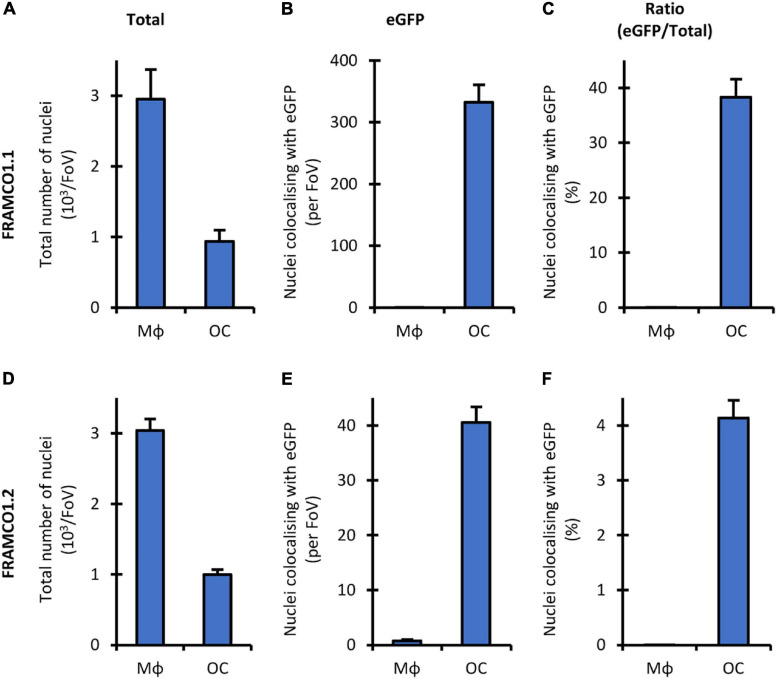
Nucleus-based quantification of the FRAMCO1.1 and 1.2 assays. Total number of nuclei **(A,D)**, nuclei localized within above-threshold eGFP^+^ objects **(B,E)** and the latter value as percentage of the former **(C,F)** in Hoechst-stained Ctsk-Cre/mTmG mono- (FRAMCO1.1, **A–C**) or Ctsk-Cre + mTmG co-cultures (FRAMCO1.2, **D–F**) of macrophages (MΦ) or osteoclasts (OC) stained and imaged on Day 4. Mean and SEM of five independent experiments is shown.

One of the limitations of the quantification of the FRAMCO1.1 (Figure 4) and FRAMCO1.2 (Figure 8) systems is that it relies entirely on the area of red and green fluorescence signals without taking into account the number and presence of cell nuclei. To provide a partial solution for that issue, we performed the quantification of Hoechst-stained cultures at a single time point on Day 4 after RANKL addition in both the FRAMCO1.1 and FRAMCO1.2 systems. As shown in Figure 10A, the number of nuclei in Ctsk-Cre/mTmG osteoclast cultures (FRAMCO1.1 system) was substantially lower than that in parallel macrophage cultures, possibly indicating less robust proliferation under osteoclastogenic conditions. Nevertheless, a substantial number of nuclei were present within green fluorescent areas in osteoclast cultures, whereas practically no such nuclei were seen in macrophage cultures (Figure 10B). As a result, nearly 40% of the nuclei in osteoclast cultures, but practically none in macrophage cultures, were within green fluorescent cells/areas (Figure 10C). A similar pattern was also seen in Ctsk-Cre + mTmG co-cultures (FRAMCO1.2 system). The overall number of nuclei (Figure 10D) was very similar to that seen in the FRAMCO1.1 system (Figure 10A). Again, nuclei within green fluorescent areas were practically only seen in osteoclast but not macrophage cultures (Figure 10E). This has translated to ∼ 4% of the nuclei being present within green fluorescent areas in osteoclast but none in macrophage cultures (Figure 10F), which is in line with the less robust nature of the FRAMCO1.2 compared to the FRAMCO1.1 system (compare Figures 4, 8).

Taken together, determining the percentage of nuclei within green fluorescent areas appears to be a viable alternative quantification strategy for the FRAMCO1.1 and FRAMCO1.2 systems. However, a limitation of this approach is the reported toxicity of Hoechst dyes during long-term fluorescence imaging (Purschke et al., 2010), which was also apparent in our hands (data not shown). We are actively working on solving this issue by introducing genetic nuclear staining into our FRAMCO1.1 and FRAMCO1.2 systems.

### The Effect of Lisophosphatidylcholine on Osteoclast Development

The ultimate aim of our experiments is to establish a framework for highly efficient and internally controlled, readily quantifiable analysis of osteoclast development. This system could be used, among others, for the analysis of the effect of various inhibitors and drug candidates. To validate the usefulness of our system for such purposes, we tested the effect of lysophosphatidylcholine (LPC), a compound previously reported to inhibit preosteoclast fusion (Verma et al., 2018), on our FRAMCO1.1 and FRAMCO1.2 systems.

**FIGURE 11:**
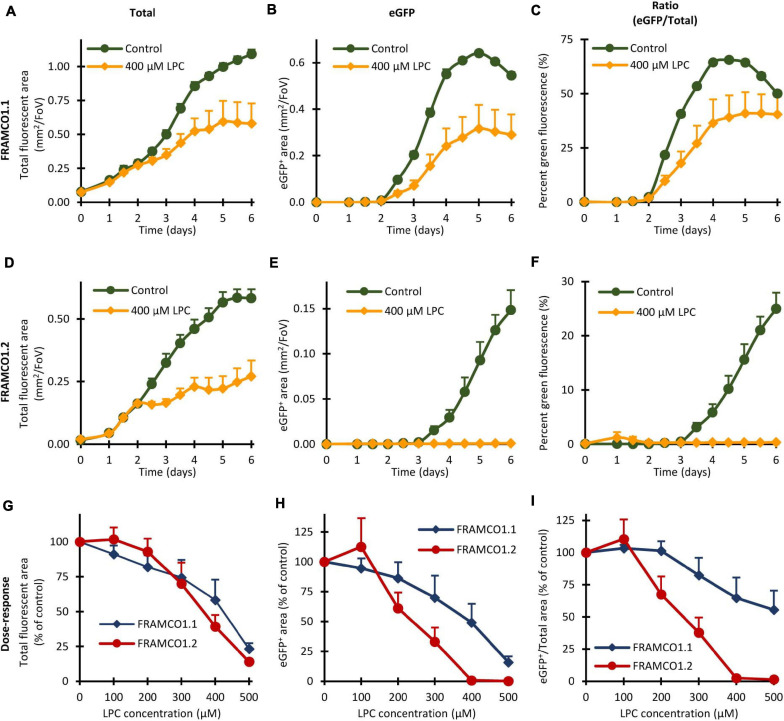
Effect of Lysophosphatidylcholine (LPC) in the FRAMCO1.1 and FRAMCO1.2 assays. **(A–F)** Time course of changes in total (tdTomato + eGFP) fluorescent area **(A,D)**, green (eGFP-positive) fluorescent area **(B,E)** or in the green area as percentage of the total fluorescent area **(C,F)** in the absence or presence of 400 μM lysophosphatidylcholine (LPC) as detected in Ctsk-Cre/mTmG (FRAMCO1.1, **A–C**) or Ctsk-Cre + mTmG (FRAMCO1.2, **D–F**) osteoclast cultures. **(G–I)** Dose-response curves of the FRAMCO1.1 and FRAMCO1.2 assays showing total fluorescent area **(G)**, green (eGFP-positive) fluorescent area **(H)** and the latter as percentage of the former **(I)** on Day 5 in the presence of the indicated LPC-concentrations. Values in **(G–I)** are shown as percentage of the controls not treated with LPC. Mean and SEM of six independent experiments is shown.

Quantification of the effect of LPC in our systems is shown in Figure 11, whereas snapshot images taken on Day 5 are included in Supplementary Figure 1. As shown in Figure 11A, 400 μM LPC partially inhibited the growth of total (red and green) fluorescent areas in Ctsk-Cre/mTmG (FRAMCO1.1) osteoclast cultures, possibly reflecting an inhibitory effect on cellular proliferation. The emergence of green fluorescence was inhibited by slightly even more, though eGFP-positive areas still developed (Figure 11B). As a result, the percentage of green fluorescence (Figure 11C) was only moderately reduced, especially at later time points. A substantially different picture was seen in Ctsk-Cre + mTmG co-cultures (FRAMCO1.2) kept under osteoclastogenic conditions. The partial inhibitory effect of LPC on apparent cell proliferation was also evident under such conditions (Figure 11D). However, while eGFP-positive cells readily emerged in control osteoclast cultures, practically no eGFP positivity could be observed in LPC-treated cultures (Figure 11E) and this also translated into the abrogation of the increase in percent green fluorescence (Figure 11F). The presence of green fluorescence and its confinement to apparently mononuclear cells in the FRAMCO1.1 but not in the FRAMCO1.2 system at 400 μM LPC concentration is also evident in the snapshot images shown in Supplementary Figure 1. We have also performed dose-response analyses in the above systems. As shown in Figure 11G, increasing LPC concentrations reduced the total fluorescence signals in both the FRAMCO1.1 and FRAMCO1.2 systems in a mostly parallel manner. Although higher doses of LPC also reduced the emergence of green fluorescence in both systems, this was substantially more pronounced in the FRAMCO1.2 than in the FRAMCO1.1 system (Figure 11H), reaching practically complete inhibition in the FRAMCO1.2 system at approximately 400 μM. This can also be seen in the snapshot images shown in Supplementary Figure 1. As a result, the percent green fluorescence was only moderately reduced in the FRAMCO1.1 but practically reached zero in the FRAMCO1.2 system (Figure 11I) at higher LPC doses.

Taken together, the above experiments confirm an inhibitory effect of LPC on preosteoclast fusion, but they also reveal additional partial inhibitory effects on osteoclast-specific gene expression and, likely, proliferation of precursor cells.

## Discussion

We have developed two separate assays that allow the fluorescence-based real-time analysis of osteoclast-specific gene expression (FRAMCO 1.1; Figures 2–5) and intercellular fusion of osteoclast-lineage cells (FRAMCO 1.2; Figures 6–9) during *in vitro* cultures. Both assays rely on red-to-green fluorescence conversion due to the recombination of the mTmG transgene by the Cre recombinase driven by the osteoclast-specific *Ctsk* promoter. The assays allow the prolonged real-time analysis of osteoclast development and are suitable for automated quantification, thereby overcoming the main limitations of the widely used TRAP staining procedure. The two assays also allow the parallel comparison of the osteoclast-specific gene expression and the intercellular fusion components of osteoclast development. Since the assays can be readily performed in multi-well plate formats, they can be adapted to higher-throughput screening of drug candidate molecules. Though we are aware that our methodological study does not provide substantial novel information on osteoclast biology, we hope that the experimental approaches described here will contribute to such discoveries in the future.

We have tested a number of control conditions to ensure that our assays do indeed reflect the transcriptional activity and fusion of osteoclasts. The facts that emergence of green fluorescence required both Cre expression and the mTmG transgene, and that no green fluorescence was observed in parallel macrophage cultures (Figures 2, 4, 6, 8), confirmed the suitability of our assay for analysis of osteoclast development. Additional studies on DNA, mRNA and bulk protein level (Figures 5, 9) also confirmed the basic principle of our assays and its reliance on osteoclast-specific gene expression and intercellular fusion.

Our results may also provide some insight into the kinetics of (*in vitro*) osteoclast development. Analysis of the time-course of osteoclast-specific gene expression (Figure 4F) revealed that it mainly occurred around Day 3 under osteoclastogenic conditions (though variation between individual cultures was seen). In contrast, the fusion process (Figure 8F) mainly occurred around Days 4–5. It is also striking that green fluorescence emerged in apparently mononuclear cells (supposedly mononuclear preosteoclasts) in the FRAMCO1.1 system, whereas eGFP expression was confined to larger, supposedly multinuclear cells in FRAMCO1.2. Though care should be taken when interpreting data from the expression of a single gene (*Ctsk* in FRAMCO1.1) and a not yet fully established fusion assay (FRAMCO1.2), our results would be in line with a differentiation pathway where osteoclast-specific gene expression changes mostly precede preosteoclast fusion, at least in *in vitro* assays triggered by recombinant cytokines. It should also be mentioned that the FRAMCO1.2 system is expected not only to detect the first fusion events but also consecutive fusion steps through the inflow of new Cre-expressing (Ctsk-Cre) and red-to-green conversion-capable (mTmG) nuclear material, as well as through the tentatively enhanced spreading of gradually enlarging multinucleated cells. These processes may be responsible for the accelerated increase of green fluorescent areas around Days 4.5–5 in the FRAMCO1.2 system (Figure 8E). Nevertheless, the relationship between actual fusion and our area-based quantification is likely not entirely linear. Further steps (such as improved long-term nucleus-based quantification; see below) need to be taken to ensure a more linear quantification of the fusion process. The presence of green fluorescence signals declined by Day 6 in both assays, likely reflecting the apoptosis of primary osteoclasts during prolonged *in vitro* culture.

Further comparison also revealed additional differences between our two assays beyond their different kinetics. Our osteoclast-specific gene expression assay (FRAMCO1.1) was clearly more robust than the fusion assay (FRAMCO1.2). This is indicated by higher level of green fluorescence (compare Figures 4F vs. 8F, 10C vs. 10F), higher level of mTmG allele cleavage at the DNA level (compare Figures 5A, 9A), and a much more robust signal in eGFP immunoblots (compare Figures 5D,E, 9D,E). Another obvious difference is that green fluorescence can be observed even in mononuclear cells in FRAMCO1.1 (Figures 2, 3), whereas practically no green fluorescence could be observed in mononuclear cells in the FRAMCO1.2 assay (Figures 6, 7). This observation likely reflects the fact that osteoclast-specific gene expression precedes, and is a prerequisite of, intercellular fusion in osteoclast cultures.

There have been a number of prior approaches to test osteoclast development by fluorescence-based tools. Transgenic expression of tdTomato driven by the *Acp5* (TRAP) promoter (TRAP-tdTomato mutation) (Kikuta et al., 2013) is in principle similar to our fluorescence-based osteoclast-specific gene expression assay. Nevertheless, the two assays use different osteoclast-related promoters (transgenic *Acp5* vs. endogenous *Ctsk*) and different labels (tdTomato vs. eGFP) to visualize osteoclasts, and require different (single or double) number of steps to trigger fluorescence, all of which may influence the osteoclast-related signals obtained. In addition, a clear advantage of our system is the basal red fluorescence of cells that have not undergone osteoclast-specific gene expression changes, providing a highly useful internal reference during automated quantification (Figures 4, 8). Regarding fluorescence-based preosteoclast fusion assays, most such assays tested the fusion of two differentially labeled cells, using either chemical (Verma et al., 2018) or genetic (Levaot et al., 2015) labeling tools. Though that approach allows the tracking of the differentially labeled cells during the fusion process, it has substantial limitations, such as the complex computational approaches required to exclude non-fused but overlapping cells, as well as the closely related high background noise, especially when higher cell concentrations are used (which may be required for optimal fusion). In contrast, our fusion assay relies on the emergence of green fluorescence only in cells that have undergone the fusion process, leading to a very good signal-to-noise ratio (Figure 8). Therefore we believe that, at least for the overall analysis of intercellular fusion, our assay is superior to those based on the co-localization of labels originating from two differentially labeled cells. It should also be noted that a prior study utilized a two-component retroviral packaging system to test fusion of osteoclast-related cells (Kondo et al., 2004); however, the complicated readout (viral titer measurement) and the lack of a fluorescence signal did not allow the real-time analysis of the fusion process in that assay.

Our fluorescence-based assays generate a very large amount of detailed high-content imaging information, with related challenges of image data processing. Our main approach was to use area-based calculations, assigning red or green fluorescence to pixels that reach a certain intensity above local background, and performing all further processing steps based on areas calculated from those binary images. Potential disadvantages of this approach are that it does not differentiate between dim and bright fluorescence above the threshold level or that it is also sensitive to changes in cell spreading without changes in overall amount of eGFP. We have not yet been able to solve those potential concerns without generating even more problems. Another possible approach would be to count the number of nuclei and assign red or green fluorescence to each nucleus based on fluorescence in the surrounding area. We have performed such calculation for a single time point in Figure 10. However, in agreement with previous reports (Purschke et al., 2010), the Hoechst dye used for those experiments interfered with the long-term cultures in our hands (data not shown) and therefore could not be used to follow osteoclast development during a prolonged culture period. We are actively working on incorporating genetic nuclear staining into our assay systems to allow nucleus-based real-time quantification in future protocols.

Another challenging issue is the acquisition and processing of high temporal resolution time-lapse image series (videos) from cell cultures over prolonged periods (several days). Though there are a few studies reporting such long-term fluorescence-based live cell imaging of osteoclast development (Levaot et al., 2015; Takegahara et al., 2016), such experiments are still scarce in the literature, especially using primary cells in a multiplate format. Since the built-in incubator of our confocal imaging system seemed not to be entirely optimized for prolonged culture of sensitive primary cells or imaging a large number of parallel samples, we performed most of our experiments by keeping the cells in a regular tissue culture incubator and moving them to our imaging station only briefly at the indicated time points. Nevertheless, we have also been able to generate time-lapse videos from a limited number of cultures, demonstrating the usefulness of our system for real-time video microscopy. Taken together, the genetic approach described here places demanding requirements on the imaging system used to record fluorescence changes in such cultures.

One of the potential uses of our assays is the screening of drug libraries on osteoclast cultures. To validate this approach, we have tested the effect of LPC on the FRAMCO1.1 and FRAMCO1.2 systems (Figure 11). While our results confirmed prior reports showing an inhibitory effect of LPC on preosteoclast fusion (Verma et al., 2018), it also revealed additional effects of LPC on osteoclast-specific gene expression and, likely, the proliferation of the cells (Figure 11). Those results exemplify the depth of details that can be obtained using the FRAMCO1.1 and FRAMCO1.2 systems. Additional questions that may arise in drug screening experiments are the specificity and sensitivity of our approach. The inclusion of parallel macrophage cultures throughout our study and the lack of any sign of eGFP expression in those cultures revealed a very high specificity for osteoclasts. Additional experiments aiming to test the sensitivity of our assays revealed that they are able to detect eGFP signals caused by as low as 1–2 ng/ml RANKL, with an apparently higher sensitivity of the FRAMCO1.1 than the FRAMCO1.2 system (data not shown).

It should also be noted that a parallel study using Ctsk-Cre and mTmG for the visualization of osteoclasts was reported during the course of our experiments (Li et al., 2018). In that complex study, various aspects of osteoclast biology were addressed using Ctsk-Cre and mTmG mutations present either in the same cell or in two different cell populations. Using those approaches, *in vitro* and *in vivo* emergence of cells showing osteoclast-specific gene expression, *in vitro* emergence of fused cells, the effect of PTH and osteoblasts/osteocytes on the fusion process and FACS-based sorting and gene expression of the fused cells was tested. Though our own experiments were initiated before the publication of the aforementioned work, we clearly admit the priority of publication of that study and that our work is based on a conceptually identical approach. Nevertheless, we believe that the more focused nature of our experiments, the clear distinction between the osteoclast-specific gene expression and intercellular fusion assays, the detailed controls used, the careful automated quantification suitable for high-throughput studies, the real-time video microscopy experiments performed and the analysis of non-fluorescence-based readouts provide highly valuable information and tools for the fluorescence-based analysis of osteoclast development. It should also be mentioned that the Li et al. (2018) study suggested the existence of two distinct populations of post-fusion osteoclasts based on the presence or absence of tdTomato-derived red fluorescence. Though we have also observed dual fluorescent (red and green) cells in both of our culture systems, we have not attempted to isolate those cells and therefore cannot speculate whether they represent a unique cell population or merely an intermediate stage in red-to-green conversion.

Though our assays proved to be suitable for the real-time analysis of osteoclast development, there is room for further improvement of our approaches. The relatively weak fusion-induced eGFP fluorescence may be improved by using other (e.g., Vav-Cre, TRAP-Cre, LysM-Cre, or CX3CR1-Cre) mouse strains which begin expressing the Cre recombinase at earlier stages of precursor differentiation or provide a stronger Cre expression, even though care should be taken to maintain the specificity of the assay for osteoclasts. Our assays may be combined with genetic labeling of nuclei, allowing the parallel visualization of nuclei without using the potentially cytotoxic Hoechst dye. Combining the biochemical differentiation and fusion assays into the same single cell cultures would allow the spatiotemporal comparison of the dynamics of the two processes. Applying the osteoclast-specific gene expression and/or the fusion assays to live mice could improve the *in vivo* analysis of osteoclast development.

We believe that the experiments presented in this report represent a major step toward the fluorescence high-content imaging-based, high-throughput analysis of osteoclast development, as well as a good starting point for further follow-up directions.

## Data Availability Statement

The raw data supporting the conclusions of this article will be made available by the authors, without undue reservation.

## Ethics Statement

The animal study was reviewed and approved by the Animal Experimentation Review Board of Semmelweis University.

## Author Contributions

ÁP, DG, and AM conceived the study. ÁP and AM designed the experiments, analyzed and interpreted the data, and wrote the manuscript. ÁP performed most of the experiments. SN performed preliminary experiments. JF, ZJ, and DG provided intellectual advice. AM supervised the project. All authors contributed to the article and approved the submitted version.

## Conflict of Interest

The authors declare that the research was conducted in the absence of any commercial or financial relationships that could be construed as a potential conflict of interest.
