# Supplementary material for: Fluorescence-Based Real-Time Analysis of Osteoclast Development
Source: Front Cell Dev Biol. 2021 Jul 13;9:657935. doi: 10.3389/fcell.2021.657935 (PMC8314002; doi:10.3389/fcell.2021.657935)
Supplement: Supplementary file 4 [file Data_Sheet_1.PDF]

Frontiers in Cell and Developmental Biology
Research Topic: Developmental Biology and Regulation of Osteoclasts

Supplementary Material

Fluorescence-based real-time analysis of osteoclast development

Áron Pánczél, Simon P. Nagy, János Farkas, Zoltán Jakus, Dávid S. Győri and Attila Mócsai

Department of Physiology, Semmelweis University School of Medicine, Budapest, Hungary

Supplementary Video 1

Real-time time-lapse imaging in the FRAMCO1.1 system

Videos are compiled in a 2×3 format with the three movies in the upper row showing the red (labeled as “tdTomato”) and the green (labeled as “eGFP”) channels and their composite (denoted as “Merge”) in Ctsk-Cre/mTmG macrophage cultures, while the lower row depicts the same channels for Ctsk-Cre/mTmG osteoclasts. The scale bar in the upper left corner in both rows corresponds to 100 µm and applies for every channel. The timestamp in the lower right corner of both rows shows the time passed after RANKL was first added to osteoclasts in an hhh:mm format. Videos are representative of 2 independent experiments.

Supplementary Video 2

Real-time time-lapse imaging in the FRAMCO1.2 system

Videos are compiled in a 2×3 format with the three movies in the upper row showing the red (labeled as “tdTomato”) and the green (labeled as “eGFP”) channels and their composite (denoted as “Merge”) in Ctsk-Cre + mTmG macrophage co-cultures, while the lower row depicts the same channels for Ctsk-Cre + mTmG osteoclasts. The scale bar in the upper left corner in both rows corresponds to 100 µm and applies for every movie. The timestamp in the lower right corner of both rows shows the time passed after RANKL was first added to osteoclasts in an hhh:mm format. Note that this video starts at 48 hours after RANKL addition just like Supplementary Video 1. Videos are representative of 2 independent experiments.

Supplementary Video 3

Real-time time-lapse imaging in the FRAMCO1.2 system

Videos are compiled in a 2×3 format with the three movies in the upper row showing the red (labeled as “tdTomato”) and the green (labeled as “eGFP”) channels and their composite (denoted as “Merge”) in Ctsk-Cre + mTmG macrophage co-cultures, while the lower row depicts the same channels for Ctsk-Cre + mTmG osteoclasts. The scale bar in the upper left corner in both rows corresponds to 100 µm and applies for every channel. The timestamp in the lower right corner of both rows shows the time passed after RANKL was first added to osteoclasts in an hhh:mm format. Note that this video starts at 60 hours after RANKL addition unlike Supplementary Video 2. Videos are representative of 2 independent experiments.

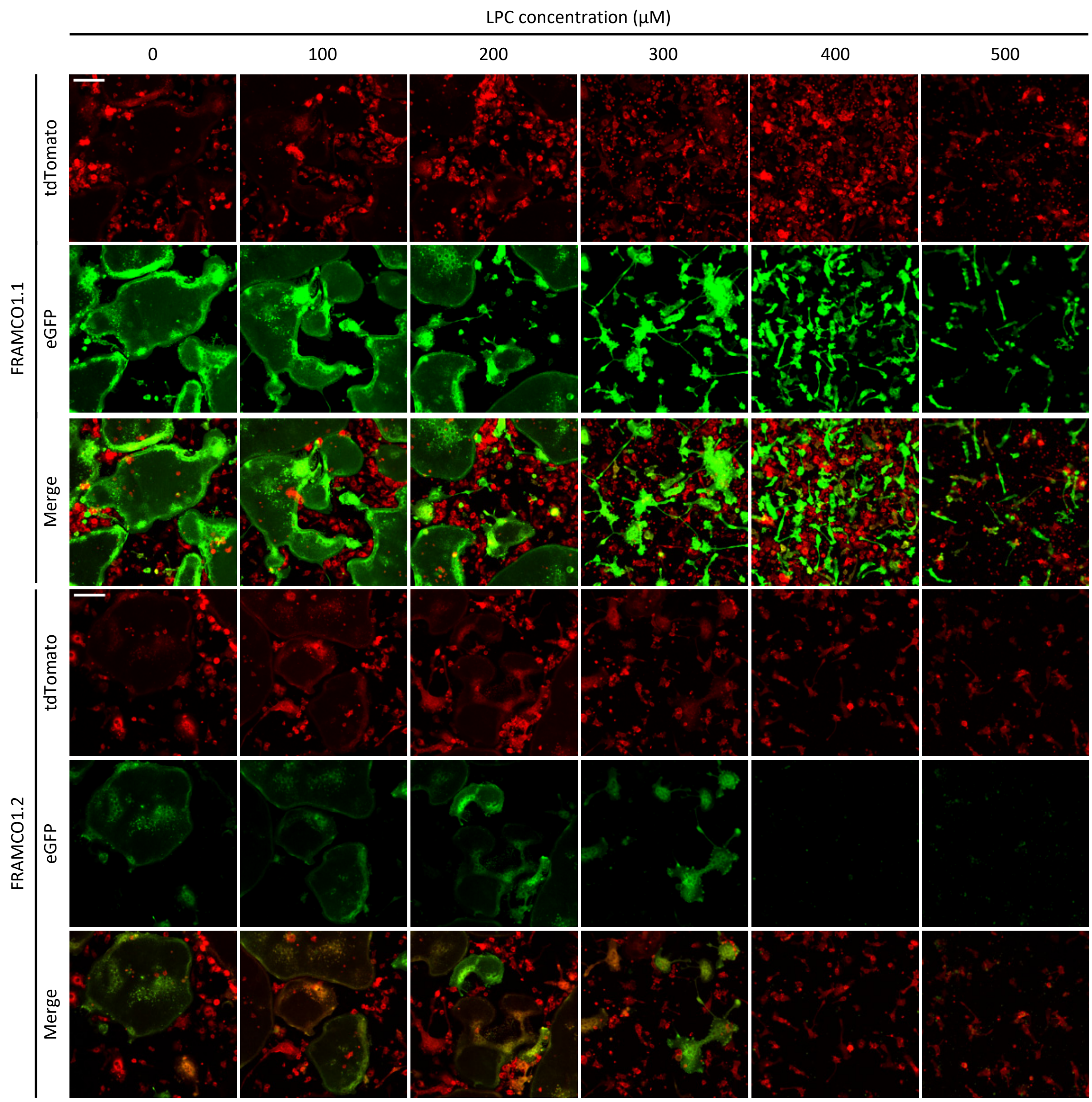

Supplementary Figure 1

Representative images of lysophosphatidylcholine-treated osteoclast cultures

Images of Ctsk-Cre/mTmG (FRAMCO1.1) osteoclast mono- and Ctsk-Cre + mTmG (FRAMCO1.2) osteoclast co-cultures treated with the indicated lysophosphatidylcholine (LPC) concentrations, taken on Day 5 (where Day 0 is the time RANKL was first added to the cultures). Rows 1 and 4 show the green, 2 and 5 the red channel, 3 and 6 depict composite images of the above two channels. Scale bar corresponds to 100 μm and applies to all images. Images are representative of 6 independent experiments.
